# Exploring Practice Patterns of Pediatric Telerehabilitation During COVID-19: A Survey Study

**DOI:** 10.1089/tmj.2021.0506

**Published:** 2022-10-07

**Authors:** Maninderjit Kaur, Emily Z. Eddy, Devashish Tiwari

**Affiliations:** ^1^ Department of Physical Therapy, MGH Institute of Health Professions, Charlestown, Massachusetts, USA.; ^2^ Department of Occupational Therapy, MGH Institute of Health Professions, Charlestown, Massachusetts, USA.; ^3^ Department of Physical Therapy, Simmons University, Boston, Massachusetts, USA.

**Keywords:** telerehabilitation, pediatric, COVID-19, physical therapy, occupational therapy

## Abstract

**Background and Objective:**

Sudden transition to telerehabilitation during the coronavirus disease 2019 (COVID-19) pandemic was challenging for pediatric therapists, including physical therapists and occupational therapists, due to lack of prior experience and knowledge. The primary goal of the current study was to survey the pediatric therapists regarding the practice trends and specific challenges/strengths of delivering telerehabilitation during the pandemic.

**Materials and Methods:**

An electronic survey was developed by the research term and validated through cognitive interviews with three pediatric therapists. A total of 107 therapists completed the survey. Descriptive statistics were used to summarize the trends for the survey questions.

**Results:**

The majority of therapists (92.5%) reported no prior experience with telerehabilitation. When comparing telerehabilitation with standard-of-care, the therapists reported similar session durations and frequencies, but greater caregiver-initiated cancellations of telerehabilitation sessions. Furthermore, a greater percentage of therapists modified the intervention activities compared with assessments, which impacted therapists' perceptions about quality of telerehabilitation as a greater percentage of therapists expressed confidence in treating children compared with assessing children virtually. One of the commonly reported telerehabilitation challenges was reduced virtual engagement of children, and strength was better assessment of home environment. Lastly, a greater percentage of therapists relied on consultations and fewer therapists used empirical evidence to guide their delivery of virtual care.

**Conclusions:**

Telerehabilitation is a cost-effective health care model that offers remote accessibility and flexible scheduling. However, several limitations in the current pediatric telerehabilitation model, including lack of teleassessments and empirical evidence, could limit post-COVID use of telerehabilitation.

## Introduction

The outbreak of the coronavirus disease 2019 (COVID-19) resulted in a widespread impact on the delivery of pediatric rehabilitation, with several clinical settings rapidly transitioning to telerehabilitation. Telerehabilitation refers to virtual delivery of clinical rehabilitation services, including evaluation, diagnosis, and treatment. Telehealth, on the contrary, is an umbrella term for both nonclinical and clinical services.^1^ Telerehabilitation utilizes telecommunication technologies, including real-time audio and videoconferencing, between patients and health care providers synchronously. Similarly, recorded videos and images, online resources, and e-mails could be asynchronous forms of data sharing.^2,3^

Telerehabilitation became an important and essential strategy to deliver care to children during the pandemic. An international survey of physical therapists (PTs) from 76 countries indicated only 4% of the therapists using telerehabilitation pre-COVID (August 2019). However, when resurveyed post-COVID (May 2020), 70% of the therapists reported using telerehabilitation to deliver care in their work setting.^4,5^

Telerehabilitation is not a newer concept and its use has been documented in the medicine field since the 1950s.^6^ The World Health Organization has endorsed the efficacy of telerehabilitation as a service delivery model for rehabilitation professionals.^7^ Pediatric telerehabilitation provides novel opportunities due to its cost-effectiveness, remote accessibility, and flexible scheduling options for caregivers and families.^2,3^

A systematic review and meta-analysis indicated that telerehabilitation is equivalent to standard in-person care for improving physical function of individuals with musculoskeletal disorders, and a hybrid model of telerehabilitation and in-person care is superior to in-person care alone.^8^ Moreover, recent evidence for telerehabilitation services (i.e., physical therapy, occupational therapy, and speech therapy) delivered during the COVID-19 indicated high patient-reported satisfaction and value in the future use of telerehabilitation services.^9^

However, the sudden transition of pediatric rehabilitation services to telerehabilitation during COVID-19 could be challenging for PTs and occupational therapists (OTs) not only due to the problems related to service delivery (e.g., legal and licensing issues, technical difficulties, and security and privacy issues) but also due to those related to patient engagement.^2,10,11^ Pediatric PTs and OTs are challenged with maintaining child's interest while remotely performing movement rehabilitation that typically requires extensive hands-on contact.^2,10,11^

Some other barriers include ensuring caregiver accessibility, and legal aspects and assessment of readiness level in terms of using technology.^5,12^ Furthermore, there is a significant lack of rigorous research on the practice trends, educational resources, and operational guidelines for delivering telerehabilitation in children.^13^ Lack of knowledge in this regard may impede the quality of care delivered to children compared with the standard in-person care.

Given the lack of evidence and sudden transition to telerehabilitation during the pandemic, the primary goal of the current study was to explore the telerehabilitation trends and practice patterns followed by pediatric PTs and OTs during the COVID-19. In addition, the study sought to describe the perceptions, strengths, and challenges faced by pediatric PTs and OTs using telerehabilitation. To accomplish the study goals, an electronic survey was circulated among the therapists with questions about therapist demographics, practice trends, perceptions about the quality of care, and strengths/challenges of telerehabilitation.

## Materials and Methods

### RESPONDENTS

Pediatric PTs, physical therapy assistants (PTAs), OTs, and occupational therapy assistants (OTAs) were recruited by advertising the study through the national and state chapters of the American Physical Therapy Association (APTA) and American Occupational Therapy Association (AOTA), social media posts, and direct e-mails to PIs academic and clinical colleagues. Snowball sampling could have occurred as respondents were encouraged to share the study information and survey links with fellow colleagues who might be interested in participating in the study.

The study was approved by the Massachusetts General & Brigham Hospitals Institutional Review Board and all respondents provided consent electronically before completing the study survey. The inclusion criteria for the study were as follows: (1) licensed PTs, PTAs, OTs, and OTAs working in any clinical setting across the United States, and (2) therapists delivering virtual care during the pandemic to children aged ≤18 years.

### SURVEY DEVELOPMENT AND VALIDATION

The research team drafted the initial set of survey questions based on the concepts identified by previous studies conducted on telehealth and process of care in pediatric physical/occupational therapy.^14–16^ Thereafter for survey validation, the initial draft was shared with three pediatric therapists with extensive clinical/and or research experience. We conducted semistructured cognitive interviews with each of the therapists virtually,^17^ two of whom were PTs from a hospital and an early intervention setting and one was an OT working in a school-based setting. The survey was modified based on the comments and suggestions from the experts. The final version of the survey comprised four sections—(1) demographics, (2) telerehabilitation practice trends, (3) clinical care during telerehabilitation, and (4) therapist perceptions (Appendix A1).

Specifically, the demographic section gathered information about the therapists (e.g., education, clinical experience, and licensing state), and clients (e.g., age and diagnosis). The second section focused on the duration/frequency of virtual sessions in comparison with standard-of-care, and the technology used during the sessions. The third section gathered information on the clinical decision-making process involved during the selection/modification of the assessments and intervention activities, strategies used to assess patient progress and interest, as well as common challenges/strengths of telerehabilitation.

The last section on therapist perceptions had multiple statements about the quality of virtual care and therapists were asked to rate their ability to assess and treat patients virtually. The survey had a mixture of open- and close-ended questions, except the last section on therapist perceptions, which required respondents to rank on a 5-level Likert scale ranging from Strongly Disagree to Strongly Agree. The survey was distributed electronically via the Research Electronic Data Capture (REDCap) online survey software. All responses were completed anonymously between December 2020 and March 2021.

### ANALYSIS

Descriptive statistics (number and percentage of responses) were used to summarize the trends for the survey questions. In terms of missing data, the number and percentage of missing responses are indicated for each question. Open-ended questions were analyzed based on the guidelines by Braun and Clarke.^18^ Two coders met and compared their initial codes for the open-ended questions. Any conflicts were mutually resolved, and a final codebook was generated. For the current study, we are reporting on the most commonly reported constructs/codes indicated by the therapists. For the Likert-scale questions, we grouped the “Strongly Agree” and “Agree” responses together, as well as the “Strongly Disagree” and “Disagree” responses (*Appendix A1*). Note: The response percentage could exceed 100 as therapists could check multiple options for a given question.

## Results

### DEMOGRAPHICS

A total of 107 therapists (47.7% PTs, 45.8% OTs, and 6.5% PTAs/OTAs) from different clinical settings and U.S. regions completed the survey (*Table 1*). The majority of therapists had a terminal clinical doctorate or master's degree (*Table 1*), with only 25.2% with a specialized clinical training/certification such as pediatric certified specialist (PCS) or certified early intervention specialist (CEIS). On average, therapists had 12.41 years (standard deviation = 10.35) of experience working with the pediatric population, ranging from 0.5 to 44 years. The majority of therapists (92.5%) reported no prior experience with telerehabilitation. In terms of client demographics, therapists reported working with clients across different age groups and diagnosis, but there were relatively fewer children seen with musculoskeletal disorders compared with neurological, developmental, or behavioral disorders (*Table 1*).

**Table 1.** Therapist and Client Demographics

PARAMETER	***n*** (%)	MISSING ***n*** (%)
Profession (*N* = 107)		
PTs	51 (47.7)	0 (0)
OTs	49 (45.8)	
PTAs/OTAs	7 (6.5)	
U.S. regions (*N* = 107)		
Northeast	76 (67.9)	0 (0)
South	15 (13.4)	
West	11 (9.8)	
Midwest	10 (8.9)	
Clinical setting (*N* = 100)		
Outpatient	57 (57)	7 (6.5)
School based	38 (38)	
Early intervention	22 (22)	
Others (including inpatient)	5 (5)	
Highest level of education (*N* = 106)		
Clinical doctorate^a^	51 (48.1)	1 (0.9)
Master's degree	39 (36.8)	
Bachelor's degree	12 (11.3)	
Associate degree	4 (3.8)	
Clients' age (in years) (*N* = 107)		
0–3	41 (38.3)	0 (0)
3–5	66 (61.7)	
6–12	83 (77.6)	
13–18	47 (43.9)	
18+	4 (3.7)	
Clients' diagnosis^b^ (*N* = 106)		
Behavioral/learning disorders	93 (87.7)	1 (0.9)
Developmental disorders	78 (73.6)	
Neurological disorders	75 (70.1)	
Musculoskeletal disorders	32 (30.2)	
Other disorders	20 (18.9)	
No. of clients per day (*N* = 105)		
0–5	29 (27.6)	2 (1.9)
6–10	66 (62.9)	
11–15	9 (8.6)	
>15	1 (1)	

^a^ Doctor of physical therapy (DPT) or doctor of occupational therapy (OTD).

^b^ Behavioral/Learning Disorders = attention-deficit/hyperactivity disorder, autism spectrum disorder, emotional, learning, sensory disorders; Developmental Disorders = global developmental delay, Down syndrome, developmental coordination disorder, prematurity; Neurological Disorders = brachial plexus injury, cerebral palsy, fragile X, muscular dystrophy, spina bifida, stroke, dyspraxia; Musculoskeletal Disorders = ligament tears, arthritis, fracture, scoliosis, torticollis; Other Disorders = visual impairment, oncology, feeding difficulty, genetic disorder, intrauterine drug exposure.

OTs, occupational therapists; PTAs/OTAs, physical therapy assistants/occupational therapy assistants; PTs, physical therapists.

### TELEREHABILITATION PRACTICE TRENDS

**Fig. 1.**
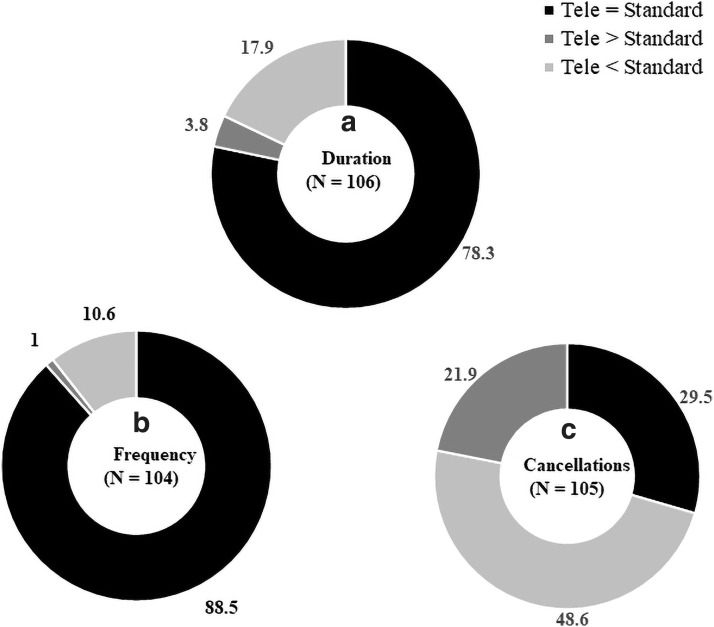
Comparison of telerehabilitation sessions **(a)** duration, **(b)** frequency, and **(c)** cancellations with the standard-of-care sessions. The percentage of respondents is indicated for each category.

The current practice trends of pediatric telerehabilitation are indicated in *Table 2*. Zoom was the most popular online platform, with a majority of the sessions done synchronously, that is, live videoconferencing with the clients compared with asynchronously, that is, use of prerecorded demo sessions or online resources (*Table 2*). The commonly reported telerehabilitation session duration was 30–60 min and frequency was ≤1 session/week (*Table 2*). Most of the therapists (78.3–88.5%) indicated that the duration/frequency of telerehabilitation sessions were similar to the standard-of-care sessions (*Fig. 1a, 1b*). However, for cancellations, 48.6% indicated greater cancellations compared with only 29.5% reporting similar cancellations for telerehabilitation and standard-of-care sessions (*Fig. 1c*).

**Table 2.** Telerehabilitation Practice Trends

PARAMETER	***n*** (%)	MISSING ***n*** (%)
Session format (*N* = 105)		
100% synchronous	82 (78.1)	2 (1.9)
% synchronous > asynchronous	22 (21)	
% synchronous < asynchronous	1 (1)	
Online platform (*N* = 105)		
Zoom	86 (81.9)	2 (1.9)
Google Meet	31 (29.5)	
FaceTime	11 (10.5)	
Doxy.me	6 (5.7)	
Teams	5 (4.8)	
Skype	3 (2.9)	
Others^a^	9 (8.6)	
Telerehabilitation duration (*N* = 105)		
<30 min	19 (18.1)	2 (1.9)
30–60 min	84 (80)	
>60 min	2 (1.9)	
Telerehabilitation frequency (*N* = 104)		
≤1 × /week	77 (74)	3 (2.8)
2–3 × /week	18 (17.3)	
≥3 × /week	1 (1)	
Other^b^	8 (7.7)	
Reasons for cancellations (*N* = 101)		
Caregiver unavailable	75 (74.3)	6 (5.6)
Poor internet connectivity	41 (40.6)	
Caregiver unwilling	32 (31.7)	
Hardware unavailability	23 (22.8)	
Child unwilling/forgetting	20 (19.8)	
Sickness (child/caregiver)	8 (7.9)	

^a^ Schoology Conferences, Videyo, WhatsApp, Teams, Go To Meeting, WebEx, Clocktree, TheraPlatform.

^b^ Variable session frequency based on client's needs.

For the open-ended question about reasons for cancellations, the majority of responses fell into the caregiver unavailability code (74.3%), followed by poor internet connectivity (40.6%), caregiver unwillingness (31.7), and hardware unavailability (22.8%) (*Table 2*).

### CLINICAL CARE DURING TELEREHABILITATION

For this section, therapists were probed about the assessments, interventions, child's engagement, and challenges/strengths of telerehabilitation (*Table 3*). A total of 58.9% therapists reported modifying the assessments and 89.6% modified the intervention activities to make them conducive for the virtual environment. For the open-ended questions about modifications, the majority of therapists focused on observational analysis of clients (53.3%) and omitting sections of standardized assessments (50%), and intervention modifications included greater reliance on family coaching (70%) and use of online games/videos (41.4%) (*Table 3*).

**Table 3.** Clinical Care During Telerehabilitation

CATEGORY	***n*** (%)	MISSING ***n*** (%)
Assessment Modifications (*N* = 60)		
Focusing on client observation	32 (53.3)	35 (36.8%)
Omitting sections of standardized test	30 (50)	
Focusing on client/caregiver report	19 (31.7)	
Assessing in-person	7 (11.7)	
Increased demonstration/cueing	5 (8.3)	
Intervention Modifications (*N* = 70)		
Heavier reliance on family coaching/assistance	49 (70)	26 (10.4)
Greater use of online games, videos	29 (41.4)	
Modifying activities/equipment (e.g., pillows as unstable surface)	12 (17.1)	
Demonstrations using toys/dummies	9 (12.9)	
Factors Impacting Child's Engagement (*N* = 65)		
Condition-related (e.g., visual impairment, attentional deficits)	39 (60)	30 (31.6)
Caregiver-related (e.g., reduced supervision)	21 (32.3)	
Technology-related (e.g., poor internet, screen fatigue)	21 (32.3)	
Environment-related (e.g., presence of other siblings, limited space)	15 (23.1)	
Age-related (e.g., infants, toddlers)	7 (10.8)	

Among all the therapists, 77.9% therapists felt that the virtual environment negatively impacted child's engagement and found children to be less engaged compared with in-person sessions. For the open-ended question about specific factors impacting child's engagement, the majority of therapists indicated condition-related (e.g., comorbidities and attentional deficits), caregiver-related (e.g., reduced supervision), and technology-related factors (e.g., screen fatigue) (*Table 3*).

**Fig. 2.**
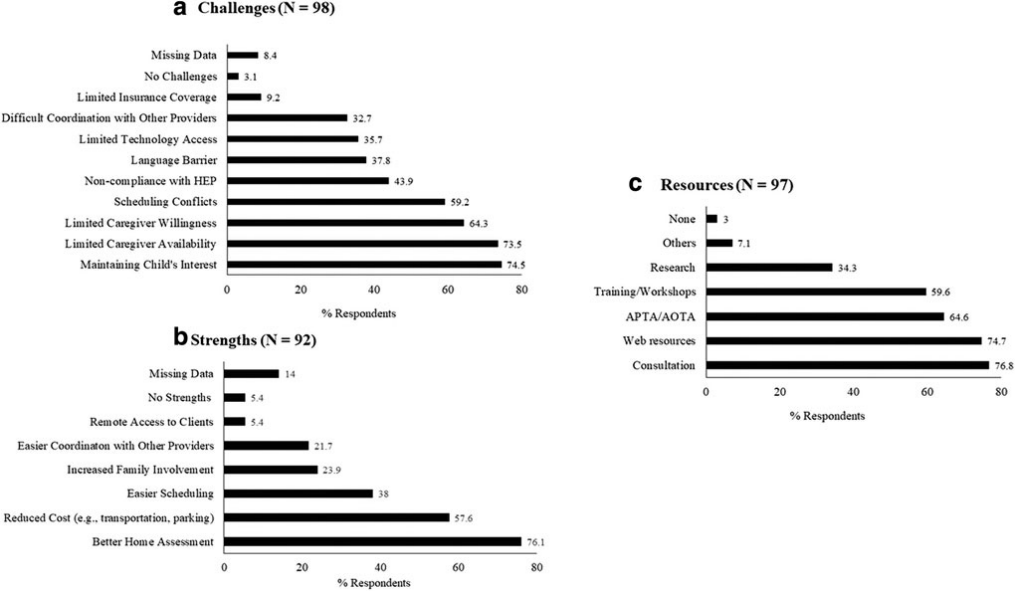
Therapist-reported **(a)** challenges, **(b)** strengths, and **(c)** resources used to guide delivery of telerehabilitation sessions during the pandemic. The percentage of respondents is indicated for each category.

Therapist-reported telerehabilitation challenges included difficulty maintaining child's interest, limited caregiver availability, and scheduling conflicts (*Fig. 2a*). On the contrary, scheduling was also identified as one of the strengths of telerehabilitation along with others such as better assessment of child's environment and reduced transportation cost (*Fig. 2b*). In terms of resources, the majority of therapists relied on professional consultations (77.3%) and web-based resources (75.3%) to guide their delivery of telerehabilitation, with fewer (34%) relying on empirical research evidence (*Fig. 2c*).

### THERAPIST PERCEPTIONS ABOUT TELEREHABILITATION

We had a mixed response for therapists' perceptions about their preparedness, knowledge, and availability of resources to conduct telerehabilitation sessions, such that 38.8–44.2% agreed and 32.7–39.8% disagreed with the statements (Statement no. 1–2, *Table 4*). Interestingly, fewer therapists (32.4%) were confident in their ability to assess/evaluate, however, the majority (70%) were confident in their ability to treat children virtually (Statement no. 3, *Table 4*). Several therapists expressed concerns with the quality of telerehabilitation such that 53.8–56.9% of therapists felt they did not deliver equivalent care or achieve all therapy goals during virtual sessions compared with standard-of-care sessions (Statement no. 4–5, *Table 4*).

**Table 4.** Therapists' Perceptions Regarding Quality of Telerehabilitation

STATEMENT	AGREE ***n*** (%)	NEUTRAL ***n*** (%)	DISAGREE ***n*** (%)	MISSING ***n*** (%)
(1) I felt fully competent and prepared while delivering telerehabilitation sessions to my clients. (*N* = 104)	46 (44.2)	24 (23.1)	34 (32.7)	3 (2.8)
(2) I had all the basic knowledge and resources (e.g., operational guidelines, research support) to successfully conduct telerehabilitation sessions. (*N* = 103)	40 (38.8)	22 (21.4)	41 (39.8)	4 (3.7)
(3) I am confident in my ability to–				
*Assess/evaluate* children during telerehabilitation sessions. (*N* = 102)	33 (32.4)	20 (19.6)	49 (48)	5 (4.7)
*Treat* children during telerehabilitation sessions. (*N* = 100)	70 (70)	20 (20)	10 (10)	7 (7)
Determine *eligibility* for future care for children during telerehabilitation sessions. (*N* = 99)	51 (51.5)	24 (24.2)	24 (24.2)	8 (7.5)
(4) I feel I was able to deliver equivalent care in telerehabilitation as during standard therapy sessions. (*N* = 102)	21 (20.1)	23 (22.5)	58 (56.9)	5 (4.7)
(5) I was able to achieve all required therapy goals during my telerehabilitation sessions. (*N* = 104)	26 (25)	22 (21.2)	56 (53.8)	3 (2.8)
(6) I feel that families/caregivers were satisfied with the delivery of telerehabilitation sessions. (*N* = 103)	67 (65)	27 (26.2)	9 (8.7)	4 (3.7)
(7) I feel the families/caregivers perceived the telerehabilitation sessions to be equally beneficial as standard in-person sessions. (*N* = 103)	22 (21.4)	40 (38.8)	41 (39.8)	4 (3.7)

Lastly, a greater percentage of therapists (65%) felt that caregivers were satisfied with the virtual sessions, but fewer therapists (21.4%) felt that caregivers perceived virtual sessions to be as beneficial as standard-of-care sessions (Statement no. 6–7, *Table 4*).

## Discussion

The primary goal of the current study was to explore the practice trends and patterns of pediatric telerehabilitation during COVID-19. Our study sample had almost equal representation of PTs and OTs, but limited participation from PTAs and OTAs. We had greater representation of the northeast region in the study probably due to the snowball sampling efforts as all study authors are from the northeast region. Similar to the current literature,^4,19^ the majority of therapists in the study expressed unfamiliarity with telerehabilitation and indicated no prior experience with the delivery of virtual care before COVID-19.

### TELEREHABILITATION VERSUS STANDARD-OF-CARE

Our survey results indicated several similarities and distinctions between telerehabilitation and standard in-person care of children with disabilities. In terms of dosage, the majority of therapists reported delivering similar frequencies and durations of telerehabilitation sessions as in-person sessions. However, there were a greater number of cancellations of telerehabilitation sessions compared with standard sessions primarily due to caregiver unavailability/unwillingness and technological challenges (*Table 2* and *Fig. 2*).

Contrary to our study, Van Houten et al. indicated that the parental involvement and compliance drastically increased when a pediatric asthma clinic transitioned to telehealth during the pandemic.^20^ Specifically, the no-shows decreased from 36% to 7.9–18% and parents expressed greater satisfaction with the newer telehealth model due to easier access, reduced cost, and time commitment. One of the reasons for this contrasting result could be the need for extensive physical contact and hands-on care required during regular PT/OT sessions, which are harder to replicate in a virtual environment.

Another important factor contributing toward compliance and acceptance of telehealth is digital literacy, that is, the ability to use information and communication technologies. In a pediatric cardiology unit, the digital literacy of the parents was directly correlated to the acceptability/usability of telehealth, such that parents who reported regular use of videoconferencing software such as Skype found telehealth to be a useful and reliable means of health care delivery for their child.^21^

Although, in our study, we did not collect information about parent demographics including digital literacy, it could have resulted in greater caregiver-initiated cancellations of the telerehabilitation sessions. Successful delivery and use of telerehabilitation in the pediatric population is dependent on caregiver involvement and engagement during the sessions,^12^ and therefore, it is imperative to provide adequate education and training to caregivers directly involved in the medical and rehabilitative care of the child.

### TELEREHABILITATION ASSESSMENTS AND INTERVENTIONS

Our survey results indicated that several therapists modified the assessments and intervention activities to better fit the needs of the virtual sessions. However, a greater percentage of therapists modified the intervention activities compared with the assessments probably because a majority of the current pediatric assessment/measurement tools are not validated for virtual use and have a standardized method of administration and scoring that cannot be easily transitioned to a virtual environment.

Instead, intervention activities are frequently modified by therapists based on the specific needs of the child and environment, and hence, the therapists were relatively less challenged by the need to modify interventions for web-based delivery of services. This was also reflected in therapists' perceptions about the quality of telerehabilitation sessions as the majority of therapists were confident in their ability to treat virtually compared with their ability to assess virtually (*Table 4*).

With regard to assessment, observations and client/caregiver reports were among the commonly reported methods to determine eligibility and initial assessments, and to monitor the progress of children. Similar to our study, a systematic review on pediatric telehealth services reported greater use of parent satisfaction outcome measures and fewer use of child-specific measures to assess the efficacy of web-based OT, PT, and speech therapy.^22^ Moreover, the studies reporting on child-specific outcomes used a variety of stand-alone tests such as grip and pinch strength, handwriting assessment, with limited use of standardized gross and fine motor tests.^22^ Some of our survey respondents used standardized tests by omitting some sections of the test that were not conducive to the virtual environment. This is usually not advisable and raises an important question about the validity/reliability of tests.

The PT and OT professional organizations have issued some guidelines related to assessment of children in the virtual environment. Specifically, the Academy of Pediatric Physical Therapy (APPT) advises clinicians to be cautious while using standardized assessments such as the Peabody Developmental Motor Scales—2nd Edition (PDMS-2) and the Bruininks/Oseretsky Test of Motor Proficiency—2nd Edition (BOT-2) and recommends using standardized parent-reported measures as well as a variety of assessment tools to gather reliable information about the child's motor and developmental skills.^23^

Similarly, the position article from AOTA^24^ recommends using assessments that have been tested to be reliable in a virtual environment such as The Timed Up and Go Test^25^ and The Functional Reach Test and the European Stroke Scale.^26^ Furthermore, it recommends substituting virtual assessments with an in-person visit whenever needed, for example, during wheelchair and seating assessments when accurate measurements are required. Clearly, there is a lack of valid/reliable teleassessments available to OTs and PTs while assessing the performance and tracking progress of children. Therefore, future research should focus on assessing the feasibility and validity of pediatric standardized assessments in the virtual environment.

### TELEREHABILITATION CHALLENGES, STRENGTHS, RESOURCES

The commonly reported telerehabilitation challenges in our study could be grouped into child-related and caregiver-related challenges. The most common child-related challenge was difficulty maintaining child's interest during the virtual sessions, and several factors such as co-occurring attentional deficits, reduced caregiver supervision, and distracting environment impacted child's engagement during the session (*Fig. 2* and *Table 3*).

Similarly, some of the common caregiver-related challenges were limited availability/willingness and noncompliance with the home exercise program. Virtual engagement of children and caregivers is essential for successful delivery of telerehabilitation sessions, and several strategies have been proposed in the literature to improve virtual engagement of caregivers and children.^27^ Use of interactive, play-based online games could significantly improve child's engagement and interest during the sessions. Nintendo Wii game is an example of active video games used to improve the physical fitness and motor skills (e.g., balance, coordination, agility) of children with and without disabilities.^28,29^ Furthermore, online forums and group meetings could be used to create a sense of virtual community for families to share experiences, discuss challenges, as well as connect and support each other.

Traube et al. incorporated monthly group meetings to improve virtual engagement while testing the feasibility of an online parenting program focused on child development, early detection of delays, school readiness, and prevention of abuse/neglect.^30^ Overall, child/caregiver engagement poses distinct challenges during virtual sessions and therapists should strategize use of different strategies such as interactive games, technology, and creating a virtual community to improve engagement.

Contrary to the current telerehabilitation literature,^12^ technological difficulties and reduced insurance coverage were not among the top challenges in our study. This could be attributed to the time line of our survey circulation, which occurred between December 2020 and March 2021. Perhaps at this time, the therapists were much more comfortable with the technology compared with the start of the pandemic. Moreover, therapists could have been benefited by the frequent improvements in the functionality of some of the commonly used online platforms such as Zoom (https://www.zoom.us/).

In terms of telerehabilitation strengths, 76% of the therapists reported improved ability to assess the child's home environment. McCue et al. stated that one of the biggest strengths of telerehabilitation is access to client's natural environment apart from traditional benefits such as reduced cost and increased access.^31^ Specifically, for children, it is sometimes challenging to generalize new behaviors and skills learnt in the clinic/school to their home environments. On the contrary, telerehabilitation offers the unique opportunity to assess and work with children within their natural environments. The remaining telerehabilitation strengths in our study are similar to those commonly reported in the literature such as reduced cost, easier scheduling, coordination, and remote access.^2,10,11^

Lastly, in terms of resources, the majority of therapists relied on regular consultations with their peers or supervisors, web resources, as well as guidelines from professional agencies. As expected, few therapists used research evidence to guide their delivery of telerehabilitation. Currently, there is a clear lack of research on operational guidelines and regulations for conducting virtual sessions with the pediatric population. A systematic review assessing the efficacy of pediatric telerehabilitation services (PT, OT, and speech therapy) used a rating scale ranging from Level I (most rigorous research, e.g., meta-analysis) to Level V (least rigorous research, e.g., qualitative interview study) to assess the strength of evidence. The majority of studies included in the review (75%) were Level V, with complete lack of Level I and II studies.^22^

### IMPLICATIONS AND FUTURE DIRECTIONS

A collaborative effort from all stakeholders, including health care providers, family members, and researchers, is required to sustain future use of telerehabilitation. From the family's perspective, we need a better understanding of caregivers' preferences such as familiarity with technology and perceptions about the telerehabilitation model. This is imperative to ensure caregiver compliance/engagement during the virtual sessions. From the therapists' perspective, there is need of operational guidelines to assist and standardize the delivery of telerehabilitation in the pediatric population. Based on our study results, there is a clear need of valid norm-referenced and/or criterion-referenced teleassessments that are amenable in the virtual environment.

Lastly, future researchers should focus on comparing telerehabilitation with in-person sessions, testing different telerehabilitation strategies, as well as evaluating short- and long-term maintenance of intervention effects following the telerehabilitation sessions.

### LIMITATIONS

Some of the study limitations include unknown response rate, disproportionate regional representation, and survey qualitative study. Our survey was distributed electronically through the APTA/AOTA websites, direct e-mails, and word of mouth. Therefore, we are unable to determine the number of participants who accessed and passed on the opportunity to complete the survey. We do believe that our response rate could have been impacted by survey fatigue as therapists were receiving several requests for study participation throughout the 2020 year. Second, the majority of therapists were from the northeast region and we would recommend future researchers to control for regional sampling and allow equal representation from different regions.

Last, one of the widely recognized limitations of survey studies is invalid data due to careless/inattentive responding or misinterpretation of questions. However, we believe that the cognitive interviews used in the current study tremendously improved the clarity and readability of survey questions.

## Conclusions

During the pandemic, several pediatric therapists were challenged by the sudden transition to telerehabilitation due to lack of prior training and guidelines for online therapies. Through the proposed study, we investigated the practice patterns and specific challenges to the delivery of virtual physical/occupational therapy services in the pediatric population. Our study findings indicated greater session cancellations, reduced virtual engagement, and lack of standardized teleassessments and empirical evidence. To promote and sustain postpandemic use of telerehabilitation, we recommend standardization of teleassessments and operational guidelines for the delivery and utilization of pediatric telerehabilitation.

## Appendix A1. Survey Questions: Pediatric Telerehabilitation During COVID-19

### Screening Questions

1. I currently work as a ____________.
  a) Physical therapist (PT)
  b) Physical therapy assistant (PTA)
  c) Occupational therapist (OT)
  d) Occupational therapy assistant (OTA)
  e) Other; please specify your professional role _______
2. I was involved in delivering telerehabilitation (virtual care) to the pediatric population with disabilities (0–17 years) during the COVID-19 pandemic.
  a) Yes
  b) No

If answered “Other” to Question 1 and “No” to Question 2, then automatically end survey.

### [A] Demographics

1. Which state do you currently work in? *Check all that apply*.
2. Which clinical settings do you work in? *Check all that apply*.
  a) Outpatient
  b) Inpatient
  c) School based
  d) Early intervention
  e) Other; please specify __________________
3. How long have you been working as a PT/PTA or OT/OTA? _____years
4. How long have you been working with the pediatric population as a PT/PTA or OT/OTA? _______years
5. What is your highest level of education?
  a) Associate
  b) Bachelor's
  c) Master's
  d) DPT/OTD
  e) PhD, DSc, EdD (terminal doctorate)
  f) Other; please specify ________________
6. Do you have any specialized training in pediatric clinical care?
  a) PCS
  b) NCS/OCS
  c) BCP
  d) CEIS
  e) ATP (assistive technology professional)
  f) Other, please specify ______________
7. Did you have any experience delivering telerehabilitation to the pediatric population prior to COVID-19 pandemic?
  a) Yes; please specify your experience delivering telerehabilitation to children in months/years.
  b) No
8. Which age group do you commonly see for the telerehabilitation visits? *Check all that apply*.
  a) Birth to 3 years
  b) Preschoolers (3 to 5 years)
  c) School age (6 to 12 years)
  d) Adolescents (13 to 18 years)
  e) Other; please specify _____________
9. What are some of the common diagnoses of your clients seen for telerehabilitation. *Check all that apply*.
10. What is your current PEDIATRIC client load in a day? _________________

### [B] Telerehabilitation Practice Trends

1. What is the average duration of telerehabilitation visit for your clients?
  a) <30 minutes
  b) 30–60 minutes
  c) >60 minutes
  d) Other; please elaborate ________________________
    Follow-up: Is the average duration of virtual sessions similar to the standard in-person visits for your clients?
    a) Yes
    b) No, virtual sessions are usually shorter than regular in-person sessions
    c) No, virtual sessions are longer than regular in-person sessions
2. What is the average frequency of telerehabilitation visits for a client in a week?
  a) 1 × /week
  b) 2–3 × /week
  c) >3 × /week
  d) Other; please elaborate ________________________
    Follow-up: Is the frequency of telerehabilitation visits/week similar to the standard in-person frequency of visits?
    a) Yes
    b) No, average frequency has reduced
    c) No, average frequency has increased
3. Do you have a similar number of cancellations/rescheduling visits for telerehabilitation as during standard care of your patients?
  a) Yes
  b) No, more cancellations/rescheduling for telerehabilitation
  c) No, less cancellations/rescheduling for telerehabilitation
    Follow-up: What are some of the common reasons for cancellations/rescheduling of telerehabilitation visits (e.g., caregiver unavailability and poor internet). Please list below. ___________________________________________
4. Which online platform do you use for providing telerehabilitation? Check all that apply.
  a) Zoom
  b) Doxy.me
  c) Google Meet
  d) SimplePractice
  e) Skype
  f) Teams
  g) FaceTime
  h) Other; please specify __________________________
5. Is the platform that you utilize for providing telerehabilitation secure and in compliance with HIPAA/FERPA?
  a) Yes
  b) No
  c) Unsure
6. How do you conduct the telerehabilitation sessions with your clients?
  a) 100% synchronous (i.e., live videoconferencing with the client using Zoom, Skype, and FaceTime)
  b) 100% asynchronous (i.e., use of recorded sessions or online resources for delivering care)
  c) % synchronous > % asynchronous
  d) % synchronous < % asynchronous
  e) Other; please specify ____________________________________________

### [C] Therapists' Perceptions About Telerehabilitation

Please rate the following questions on a Likert scale from 1 to 5:

1 = Strongly Disagree, 2 = Disagree, 3 = Neutral, 4 = Agree, 5 = Strongly Agree
1. I feel fully competent and prepared in delivering telerehabilitation sessions to my patients.
2. I had all the basic resources and knowledge to successfully conduct telerehabilitation sessions.
3. a. I am confident in my ability to accurately assess/evaluate children during telerehabilitation sessions.
  b. I am confident in my ability to accurately treat during telerehabilitation sessions.
  c. I am confident in my ability to accurately determine eligibility for future care during telerehabilitation sessions.
4. I feel I was able to deliver equivalent care in telerehabilitation as during standard therapy sessions.
5. I was able to achieve all required therapy goals during my telerehabilitation sessions.
6. I feel the families/caregivers were satisfied with the delivery of the telerehabilitation sessions.
7. I feel the families/caregivers perceived the telerehabilitation sessions to be equally beneficial as standard in-person sessions.

### [D] Clinical Care and Decision-Making During Telerehabilitation

1. Did you do any modifications to your commonly used assessments/measurement tools (e.g., standardized tests, ROM, and functional assessment) to make them more conducive to the visual environment?
  a) Yes
  b) No
    Follow-up: If yes, please specify what kind of modifications were done to the common assessment tools (e.g., omitting parts of a standardized test, relying only on natural observation of child ___________________________________________
2. Did you do any modifications to your intervention activities to make them more conducive for the virtual environment?
  a) Yes
  b) No
    Follow-up: If yes, please specify what kind of modifications were done to the intervention activities (e.g., use of Bitmoji). ___________________________________________
3. Do you think children were equally engaged during telerehabilitation sessions compared with standard in-person sessions?
  a) Yes
  b) No
    Follow-up: If no, please specify some of the challenges faced while maintaining child's engagement during virtual sessions (e.g., child's inability to tolerate screen time). ___________________________________________
4. What are some of the common challenges faced by you while delivering telerehabilitation to your clients? *Check all that apply*.
  a) Insurance coverage
  b) Scheduling conflicts
  c) Maintaining child's interest
  d) Limited caregiver availability
  e) Difficulty coordinating care with colleagues or other service providers
  f) Noncompliance with home exercise program
  g) Limited access to technology/ hardware
  h) Language barrier
  i) Other; please specify ___________________________
5. What are some of the strengths of delivering care via telerehabilitation compared with standard in-person care? *Check all that apply*.
  a) Reduced cost
  b) Easier scheduling
  c) Better assessment of child's home
  d) Easier coordination with other service providers
  e) None
  f) Other; please specify __________________________
6. Please list the resources used to assist your delivery of telerehabilitation. *Check all that apply*.
  a) APTA/AOTA guidelines
  b) Research evidence
  c) Regular consultation with colleagues/supervisor
  d) Training or educational workshops
  e) Web resources including social media groups
  f) Other; please specify
  g) None
    Follow-up: Other, please specify: ___________________________________________
7. What kind of additional resources do you feel could significantly improve the future delivery of your telerehabilitation sessions? ______________________________________________
8. What are some of the changes/modifications made to your current delivery of telerehabilitation compared with earlier in the year (i.e., at the start of pandemic during February/March of 2020)? ______________________________________________
